# The SIRT1-Mediated p53 Deacetylation Pathway Modulates Apoptosis and Promotes Viral Replication in MVC-Infected Cells

**DOI:** 10.3390/pathogens15030242

**Published:** 2026-02-25

**Authors:** Yan Yan, Xiang Ren, Yishu Xiao, Fang Li, Jianhui Guo, Kai Ji, Zhiping Hei, Zhijie Zhang, Yuning Sun

**Affiliations:** 1 Department of Biochemistry and Molecular Biology, School of Basic Medical Science, Ningxia Medical University, Yinchuan 750004, China; 2 Ningxia Institute of Clinical Medicine, People’s Hospital of Ningxia Hui Autonomous Region, Ningxia Medical University, Yinchuan 750002, China; 3 Peking University First Hospital Ningxia Women and Children’s Hospital (Ningxia Hui Autonomous Region Maternal and Child Health Hospital), Yinchuan 750001, China; 4 The Third Clinical Medical College of Ningxia Medical University, People’s Hospital of Ningxia Hui Autonomous Region, Ningxia Medical University, Yinchuan 750002, China; 5 Department of Respiratory and Critical Care, General Hospital of Ningxia Medical University, Ningxia Medical University, Yinchuan 750004, China

**Keywords:** MVC, SIRT1, p53 deacetylation, apoptosis, viral replication

## Abstract

Minute virus of canines (MVC) is an autonomous canine parvovirus that causes severe pathological outcomes, including embryo mortality, spontaneous abortion, and congenital malformations in neonatal puppies. Although MVC infection has been shown to induce host cell cycle arrest and apoptosis, the underlying regulatory mechanisms that coordinate cell proliferation and control apoptotic responses during viral replication remain poorly understood. Sirtuin 1 (SIRT1) is an NAD^+^-dependent deacetylase that plays a critical role in regulating cell cycle progression, DNA damage responses, and apoptosis. However, its involvement in MVC infection has not been fully elucidated. Herein, we show that MVC infection markedly upregulates the mRNA and protein expression levels of SIRT1 in a time-dependent manner. MVC infection activates the SIRT1-p53 signaling axis and modulates the acetylation status of p53. In addition, MVC alters the subcellular distribution of SIRT1, promoting its nuclear translocation and colocalization with the viral protein VP2. Functional analyses demonstrated that pharmacological activation of SIRT1 enhanced the viability of MVC-infected WRD cells (virus-tropic cell), promoting viral replication, prolonging S-phase arrest, and reducing apoptosis. Conversely, inhibition of SIRT1 produced the opposite effects, which were closely associated with regulation of the SIRT1-p53 signaling axis. Moreover, SIRT1 knockdown accelerated apoptosis and attenuated S-phase arrest, whereas SIRT1 overexpression further strengthened S-phase retention. Collectively, our findings identify the SIRT1-p53 signaling axis as an important regulator of cell cycle progression and apoptosis during MVC infection, highlighting SIRT1 as a key host factor that supports viral replication and persistence and a potential target for antiviral intervention.

## 1. Introduction

Minute virus of canine (MVC), a typical representative of the genus *Bocaparvovirus* within the subfamily *Parvovirinae*, was first isolated from canine fecal specimens in the United States [1]. MVC infection causes a range of disorders, including viral hepatitis, acute gastroenteritis, pneumonitis, and myocarditis in puppies and immunocompromised adults, while posing a reproductive risk through mother-to-child transmission via the placenta, which can lead to embryonic or fetal mortality [2,3,4,5]. Similar to other bocaparvoviruses, MVC possesses a non-enveloped icosahedral capsid and a single-stranded DNA genome of approximately 5.4 kb. Notably, its genome contains an additional open reading frame (ORF) located between the non-structural (NS) and structural (VP) coding regions, which encodes the 21 kDa non-structural protein NP1, representing a distinctive genomic feature unique to bocaparvoviruses [6]. This special genome arrangement breaks through the conventional coding pattern of the parvovirus family, making bocaparvoviruses genomically unique with potential functional implications for viral replication and host interaction [1,7,8,9]. Taxonomically, these small non-enveloped ssDNA viruses exhibit broad host tropism, infecting humans, domestic dogs, and multiple mammalian species [1,7,10]. Recent advances in metagenomic sequencing have identified bocaviruses, which are closely related to parvoviruses, in an expanding range of animal hosts [11,12,13,14,15].

The main mechanisms of parvovirus-induced cell death exhibit remarkable viral species specificity. Apoptosis remains the predominant mode of cell death across multiple parvoviruses [16]. However, necrotic cell death has also been observed in cells infected with murine minute virus (MVM) and H-1 parvovirus (H-1PV) [17,18]. Among bocaparvoviruses, virus-induced cell death strategies appear to be virus-specific. Human bocavirus 1 (HBoV1) has been shown to induce pyroptosis in human airway epithelial cells [19], whereas bovine parvovirus (BPV) triggers classical necrosis characterized by plasma membrane rupture and cytoplasmic content release in bovine embryonic tracheal cells [20]. In studies of MVC, the Walter Reed/3873D (WRD) cell line has been established as a well-characterized in vitro infection model that exhibits strict host tropism [1]. Previous studies have shown that MVC infection of WRD cells induces pronounced cytopathic effects (CPEs), which are primarily manifested as mitochondrion-mediated apoptosis and are accompanied by p53-dependent activation of the DNA damage response [21,22]. Although substantial progress has been made in understanding MVC biology through the construction of infectious clones and studies of cellular adaptation [1,6], the core host regulatory mechanisms governing MVC genome replication remain poorly defined. A systematic elucidation of host factors involved in MVC replication is therefore critical for advancing our understanding of viral pathogenesis, developing effective antiviral strategies, and preventing viral transmission.

In general, viral survival and replication within host cells require the exploitation of host cellular factors, the hijacking of host signaling pathways, and the disruption of host antiviral defense mechanisms to facilitate viral propagation. Accordingly, host regulatory factors play an important role during viral infection. Histone deacetylases (HDACs) constitute a superfamily of enzymes that regulate gene transcription through highly conserved deacetylase domains and are generally classified into class I, class IIa, class IIb, and class IV HDACs [23]. Another major subgroup of HDACs is class III histone deacetylases, known as sirtuins, which are mammalian homologues of the yeast silent information regulator 2 (Sir2) protein and function as NAD^+^-dependent deacetylases [24]. Seven sirtuin family members (SIRT1–SIRT7) have been identified in mammals; although they share a conserved catalytic core, they differ in subcellular localization and substrate specificity [24]. Among these members, SIRT1 is the most extensively studied sirtuin and is predominantly localized in the nucleus and cytoplasm. Through its deacetylase activity, SIRT1 regulates a wide range of cellular processes, including DNA damage repair, apoptosis, ageing, stress responses, and inflammation [25]. SIRT1 exhibits antiviral effects through multiple mechanisms, including inhibition of viral lytic replication [26], suppression of transcription from viral long terminal repeats (LTRs) [27], direction of immune responses [28], and reduction in viral titer and gene expression [29]. Paradoxically, during HBV, HIV, and HPV infections, SIRT1 plays opposing roles by enhancing viral core promoter activity [30], activating viral promoters [31], and contributing to the regulation of viral replication and gene expression [32]. Despite these findings, the role of SIRT1 in parvovirus infection remains largely unexplored.

In the present study, we demonstrated that SIRT1 expression is significantly upregulated in MVC-infected cells and evaluated the effects of SIRT1 agonists and inhibitors on the course of MVC infection. In addition, modulation of SIRT1 activity was found to regulate p53 activity, thereby influencing the cytopathic effects induced by MVC infection. Collectively, our findings reveal a regulatory role for SIRT1 in MVC-induced cell death, highlight the functional importance of the SIRT1/p53 signaling pathway in this process, and further establish a mechanistic link between host cellular factors and the MVC life cycle.

## 2. Materials and Methods

### 2.1. Cells and Virus

The WRD cell line and MVC were kindly provided by Jianming Qiu of the Department of Microbiology, University of Kansas Medical Center. The COS-1 cells used in this study were preserved in our laboratory. Cells were cultivated and maintained in Dulbecco’s modified Eagle’s medium (DMEM; Cat. No. 11965092, Gibco, Grand Island, NY, USA) supplemented with 10% fetal bovine serum (FBS; Cat. No. FS201-02, TransGen Biotech, Beijing, China) and 1% penicillin–streptomycin (Cat. No. P1400, Solarbio, Beijing, China) in a humidified atmosphere of 5% CO_2_ at 37 °C. MVC was propagated in WRD cells, and the viral titer was measured using immunofluorescence assay, as previously described [33].

### 2.2. MVC Infection

WRD cells were infected with MVC at a multiplicity of infection (MOI) of 2. The virus was diluted in serum-free DMEM and then transferred to cells pre-washed with phosphate-buffered saline (PBS; Cat. No. KGL2206-500, KeyGen Biotech, Nanjing, China). For drug treatment, the viral suspension was combined with appropriately diluted drug compounds in DMEM medium and added to WRD cells. Following a 1.5 h MVC incubation period, the infection mixture was aspirated and replaced with complete DMEM medium or with drug-supplemented complete DMEM medium. The cells were then cultivated at 37 °C for the established period.

### 2.3. Plasmids and Transfection

SIRT1 and p53 DNA were PCR-amplified from WRD cells. SIRT1 was cloned into the pCMV-HA vector by restrictive endonuclease SalI/XhoI using a forward primer (5′-GACACGTCGACCATGGCGGACGACG-3′) and a reverse primer (5′-GACACCTCGAGTTATGATTTGTTTG-3′) to establish pCMV-HA-SIRT1. p53 was cloned into pCMV-Myc vector by restriction endonuclease EcoRI/XhoI using a forward primer (5′-GACACGAATTCGGATGGAGGAGTC-3′) and a reverse primer (5′-GACACCTCGAGTCAGTCTGAGTCAAG-3′) to conduct pcMV-Myc-p53. DNA sequencing and restriction digestion were applied to confirm the established plasmids. Lipofectamine 3000 reagent (Cat. No. L3000008, Invitrogen, Carlsbad, CA, USA) was used for transfection. Briefly, cells were seeded in 6-well plates or in confocal culture dishes and incubated for 12 h. Prior to transfection, the culture medium was replaced with Opti-MEM (Cat. No. 31985062, Gibco, Grand Island, NY, USA). The transfection mixture containing the two plasmids was prepared according to the manufacturer’s recommendations. Cells were incubated with the transfection mixture for 48 h before conducting downstream analyses.

### 2.4. Cell Viability

The SIRT1 activator resveratrol (Cat. No. R5010, Sigma-Aldrich, St. Louis, MO, USA) and inhibitor Ex527 (Cat. No. E7034, Sigma-Aldrich, St. Louis, MO, USA) were dissolved in dimethyl sulfoxide (DMSO; Cat. No. D2650, Sigma-Aldrich, St. Louis, MO, USA), with stock concentrations of 200 mM and 50 mM, respectively. WRD cells were seeded at a density of 3000 cells per well in a 96-well culture plate with a volume of 100 μL. After 12 h incubation, when cell confluence reached approximately 70–80%, resveratrol and Ex527 were diluted in culture medium to generate a series of concentrations and added to the cells for a 72 h incubation to identify the maximum non-toxic concentrations.

To investigate the effects of resveratrol and Ex527 on cell viability following MVC infection, cells were infected with MVC at MOIs of 0.2, 0.5, 1, 2, 4, 8, and 16 in the presence of resveratrol or Ex527 for 1.5 h. The infection mixture was then removed, and the cells were washed with PBS. Subsequently, fresh culture medium containing 10% FBS with resveratrol or Ex527 was added, and the cells were incubated for 72 h.

The culture medium was replaced with 100 μL serum-free DMEM, and 10 μL CCK-8 reagent (Cat. No. FC101, TransGen Biotech, Beijing, China) was pipetted into each experimental well. The optical density (OD) of the plate was measured using a full wavelength microplate reader (Multiskan GO; Thermo Fisher Scientific, Waltham, MA, USA) at 450 nm following a 4 h incubation in the incubator. Cell viability was determined using the following formula: (OD450 infected − OD450 blank)/(OD450 uninfected − OD450 blank) × 100%.

### 2.5. RNA Extraction and RT-qPCR

Cells were washed with PBS, and total RNA was isolated using Trizol reagent (Cat. No. 15596026, Invitrogen, Carlsbad, CA, USA) according to the manufacturer’s instructions. RNA content was measured using a NanoDrop 2000 spectrophotometer (Thermo Fisher Scientific, Waltham, MA, USA), and 1 μg of the quantified total RNA was used for reverse transcription. cDNA was synthesized using TransScript^®^ One-Step GenomicDNA Removal and Complementary DNA (cDNA) Synthesis SuperMix (Cat. No. AT311, TransGen Biotech, Beijing, China). SYBR Green-based relative fluorescence quantitative PCR was performed using the TransStart^®^ Top Green qPCR SuperMix (Cat. No. AQ131, TransGen Biotech, Beijing, China) according to the manufacturer’s instructions. The synthesized cDNA samples were diluted 10-fold and used as templates. Primer sequences were designed as follows: SIRT1 (forward: 5′-CAGTTCCAACCATCTCTCTGTC-3′; reverse: 5′-GCAACCTGTTCCAGTGTATCT-3′); p53 (forward: 5′-CGAGGTTGGCTCTGACTATAC-3′; reverse: 5′-GAGTCTTCCAGGGTGATGATAG-3′); and GAPDH (forward: 5′-GCTGAGTATGTTGTGGAGT-3′; reverse: 5′-GCAGAAGGAGCAGAGATG-3′). The 2-Delta Ct method was used to compare the expression levels of the investigated genes with those of the endogenous control gene GAPDH in each sample.

### 2.6. Viral DNA Preparation and Quantitation

Low-molecular-weight DNA (Hirt DNA) was extracted from MVC-infected WRD cells as described previously to evaluate intracellular viral DNA copies [6]. The culture medium was harvested separately to confirm the extracellular viral DNA copies. Subsequently, viral DNA copies were determined using absolute fluorescence quantitative PCR. The infectious clone pI-MVC was diluted 10 times in proportion to a series of concentrations as the standard sample concentration. qRT-PCR was conducted as previously described. A standard curve was established using the Ct values and dilutions of pI-MVC, which was used to quantify the MVC copies.

### 2.7. Western Blotting

Total cellular proteins were isolated using a commercial protein extraction kit (Cat. No. KGB5303, KeyGen Biotech, Nanjing, China). Cells were harvested and lysed in lysis buffer supplemented with protease and phosphatase inhibitors with shaking at 4 °C for 30 min. Homogenized samples were centrifuged at 14,000 rpm for 15 min, and the cleared supernatants (containing the proteins) were transferred to new tubes. A Bicinchoninic Acid (BCA) protein assay kit (Cat. No. KGP903, KeyGen Biotech, Nanjing, China) was used to determine the concentrations of whole-cell protein extracts. The equalized proteins were subjected to SDS-PAGE and transferred to PVDF membranes (Ca. No. 0000229835, Millipore, Burlington, MA, USA). The blotted membranes were blocked in 5% skimmed milk for 1 h and incubated with the indicated primary antibodies at 4 °C overnight. After washing with TBST for 20 min, the membranes were incubated with horseradish peroxidase-conjugated anti-mouse or anti-rabbit antibodies for 1 h at room temperature. The membranes were then washed with TBST 4 times, each time for 5 min. An enhanced chemiluminescence (ECL) substrate (Ca. No. SW181-01, SEVEN Biotech, Beijing, China) was used to visualize the protein bands, and images were acquired using an Azure 300 image system (San Jose, CA, USA). The relative expression level of the indicated proteins was quantified using ImageJ v1.46 (Bethesda, MD, USA). Primary antibodies employed for Western blotting are listed below: anti-acetylated p53 (K382) (rabbit pAb, MAB13552; WB: 1:500) was obtained from R&D Systems (Minneapolis, MN, USA); anti-SIRT1 (rabbit pAb, 13161-1-AP; WB: 1:500), anti-p53 (rabbit pAb, 10442-1-AP; WB: 1:1000), anti-GAPDH (rabbit pAb, 10494-1-AP, WB: 1:10,000), anti-cleaved caspase 3 (rabbit pAb, 25128-1-AP; WB: 1:500), anti-bcl 2 (rabbit pAb, 26593-1-AP; WB: 1:400), Bax (rabbit pAb, 50599-2-Ig; WB: 1:1000), p21 (rabbit pAb, 10355-1-AP; WB: 1:500), anti-HA (rabbit pAb, 51064-2-AP; WB: 1:1000; IP: 3 μg), anti-Myc (mouse mAb, 60003-2-Ig; WB: 1:1000; IP: 3 μg), and anti-Flag (mouse mAb, 66008-3-Ig; WB: 1:1000) were obtained from Proteintech (Wuhan, China); anti-NS1(rabbit pAb, 18929-1; WB: 1:1000) and anti-VP2 (rabbit pAb, 20351-1; WB: 1:1500) were generated in collaboration with Abmart Company (Shanghai, China). The HRP-linked secondary antibodies are shown below: Goat anti-mouse (ZB-2305; WB: 1:5000) and Goat anti-rabbit (ZB-2301; WB: 1:5000) were obtained from ZSGB-BIO (Beijing, China).

### 2.8. Knockdown of SIRT1 Expression

The short-hairpin RNA (shRNA) targeting SIRT1 and the negative control shRNA lentivirus were designed and synthesized by Genomeditech (Shanghai, China). The following shRNAs were engineered to specifically target SIRT1: shRNA1 (5′-GGAACCTGCCAGAGTCCAAGT-3′) and shRNA2 (5′-GCTGATGAGCCACTTGCTATC-3′). WRD cells were seeded into 6-well plates at 2 × 10^5^ cells per well and infected with either the GFP-expressing shRNA-negative control (shNC) lentivirus or SIRT1 shRNA1/shRNA2 lentivirus. Continuous puromycin treatment (2 μg/mL) was applied throughout the culture period to establish stable shRNA-expressing lines. qRT-PCR and Western blot analysis was used to confirm SIRT1 mRNA and protein knockdown in the successful transfectants, respectively. Small interfering RNA (siRNA) oligonucleotides targeting the sequences identical to those of shRNAs were synthesized by Genomeditech (Shanghai, China). The small interfering RNA (siRNA) vector sequences are presented as follows: siRNA-1 (sense 5′-GGAACCUGCCAGAGUCCAAGUtt-3′, antisense 5′-ACUUGGACUCUGGCAGGUUCCtt-3′) and siRNA-2 (sense 5′-GCUGAUGAGCCACUUGCUAUCtt-3′, antisense 5′-GAUAGCAAGUGGCUCAUCAGCtt-3′). The siRNAs (80 nM) and a nontargeting control were transfected with Lipofectamine 3000 reagent according to the manufacturer’s protocols. A 24-h incubation was performed post-transfection before proceeding to downstream assays.

### 2.9. Overexpression of SIRT1

The construction of the SIRT1 recombinant overexpression vector and its lentiviral packaging were commissioned to GeneChem Co., Ltd. (Shanghai, China). WRD cells were infected with the lentivirus containing the SIRT1-expressing vector or with an empty plasmid. After 12 h of infection, the lentivirus-containing medium was removed and replaced with fresh culture medium. Puromycin (2 μg/mL) was added to the cell culture medium to isolate cells stably expressing Flag-SIRT1.

### 2.10. Flow Cytometry

Flow cytometry analysis was performed to examine cell cycle distribution and apoptosis. For cell cycle analysis, cells were infected with MVC and harvested 24 h or 48 h post-infection (hpi). After immobilization in 75% ethanol overnight, the cells were stained using a cell cycle detection kit (Cat. No. KGA9101, KeyGen Biotech, Nanjing, China) in accordance with the directions provided by the manufacturer. DNA content analysis of the collected cells was operated by means of flow cytometry (Beckman Coulter, Palo Alto, CA, USA). Cell cycle distribution was analyzed using ModFit software v4.1 (Topsham, MA, USA).

To detect apoptosis in MVC-infected WRD cells, the Annexin V-APC and PI apoptosis detection kit (Cat. No. AP107-60, MultiSciences, Hangzhou, China) was used according to the manufacturer’s specifications. Briefly, 48 h post-infection, cells were dispersed with Accutase, washed twice with cold PBS, and collected by means of low-speed centrifugation. A total of 100 μL binding buffer supplemented with 2 μL Annexin V-APC and 4 μL Propidium Iodide (PI) was added to each sample, with incubation in the dark for 10 min. Another 200 μL of binding buffer was added after incubation, and the cells were analyzed with flow cytometry (Beckman Coulter, Palo Alto, CA, USA). The data were processed and analyzed using FlowJo v10 (BD Biosciences, Ashburn, VA, USA).

### 2.11. Confocal Microscopy

COS-1 cells were seeded in a confocal culture dish and transfected with 0.5 μg pCMV-HA-SIRT1 and 0.5 μg pCMV-Myc-p53 plasmids simultaneously. After 48 h, the cells were washed thrice with PBS, fixed with 4% paraformaldehyde for 30 min, and permeabilized with 0.25% Triton X-100 for 20 min. The cells were incubated with 5% bovine serum albumin (BSA; Cat. No. SW3015, Solarbio, Beijing, China) at 37 °C for 30 min. COS-1 cells were incubated with a 1:100 dilution of the HA-tag and Myc-tag antibody mixture at 4 °C overnight. The next day, cells were washed thrice with PBS and incubated with goat anti-rabbit Alexa fluorescent probe 594 (Cat. No. bs-0295G, Bioss, Beijing, China; IFA: 1:200) and goat anti-mouse Alexa fluorescent probe 488 (Cat. No. bs-0296G, Bioss, Beijing, China; IFA: 1:200) secondary antibodies for 1 h. A sealing reagent to resist fluorescence quenching with DAPI (Cat. No. 62248, Thermo Fisher Scientific, Waltham, MA, USA) was used to label the nuclei before detection. Immunofluorescence analysis was performed using a confocal microscope (LSM 800, Carl Zeiss, Jena, Germany). Colocalization was quantified using the Just Another Colocalization Plugin (JACoP) of ImageJ software. Pearson’s correlation coefficient (PCC) and the overlap coefficient were calculated to assess the degree of colocalization between channels.

### 2.12. Co-Immunoprecipitation

After 48 h transfection of COS-1 cells with pCMV-HA-SIRT1 and pCMV-Myc-p53 expression plasmids, cells were collected and lysed in NP-40 lysis buffer (Cat. No. P0013F, Beyotime, Shanghai, China) supplemented with protease inhibitors. The lysate was centrifuged at 12,000 rpm for 15 min. The supernatant was collected, and 10 μL of the supernatant was retained as input control. The remaining supernatant was precipitated with either anti-Myc (mouse IgG antibody as a negative control, Cat. No. B900620, Proteintech, Wuhan, China) or anti-HA antibody (rabbit IgG antibody as a negative control, Cat. No. B30011S, Abmart, Shanghai, China) at 4 °C overnight with rotation. The next day, 50 μL protein A/G agarose beads (Cat. No. sc-2003, Santa Cruz Biotech, Dallas, TX, USA) were added to the supernatant at 4 °C for 4 h to combine with the protein–antibody complexes. The beads were centrifuged and washed thrice with ice-cold PBST. The immunoprecipitates were resuspended in 30 μL PBS and boiled at 100 °C for 5 min. After centrifugation, the supernatant was collected and subjected to Western blotting to detect either HA-tagged SIRT1 or Myc-tagged p53.

### 2.13. Statistical Analysis

Data were analyzed using Prism v.8 (GraphPad, San Diego, CA, USA) and are presented as the mean ± standard deviation (SD) of at least three independent experiments. Student’s *t*-test was used to compare mean differences between variables. A value of *p <* 0.05 was considered significant.

## 3. Results

### 3.1. MVC Infection Activates Host Deacetylation Machinery Centred on SIRT1/p53

**Figure 1:**
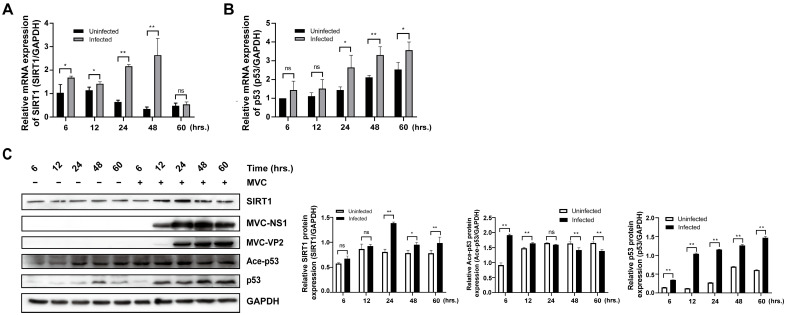
MVC infection induces SIRT1 transcription and protein expression while modulating p53 acetylation. (**A**,**B**) Relative mRNA expression levels of SIRT1 and p53 in WRD cells at the indicated time points (6–60 h) following MVC infection (MOI = 2). SIRT1 and p53 mRNA levels were quantified by means of RT-qPCR and normalized to GAPDH. (**C**) Western blot analysis of SIRT1, p53, acetylated p53 (Ace-p53), NS1, and VP2 in WRD cells at 6–60 h following MVC infection (MOI = 2). GAPDH was used as a loading control in all experiments. The relative expression of SIRT1, Ace-p53, and total p53 protein was determined by means of densitometric quantification, as shown on the right, and data are shown as averages and standard deviations from 3 independent experiments in each panel. * *p* < 0.05; ** *p* < 0.01; ns: not significant.

Previous studies have revealed that MVC infection triggers mitochondrion-mediated apoptosis and cell cycle arrest in WRD cells, with apoptotic signaling being strictly dependent on viral genome replication [21]. Since SIRT1 has been extensively implicated in the regulation of p53-dependent apoptosis and cell cycle arrest [34,35], we reasoned that SIRT1 might function as a key modulator of the cellular responses described above. We first examined the temporal dynamics of SIRT1 transcription during MVC infection. RT-qPCR analysis showed that compared with the uninfected controls, SIRT1 mRNA levels were significantly elevated at 6 hpi and continued to increase between 12 and 48 h, reaching a maximum at 48 h before declining at 60 h (Figure 1A). This expression pattern indicates that MVC infection rapidly induces the transcriptional activation of SIRT1 at the early stages of infection. To further verify the protein expression level of SIRT1, Western blot analysis was performed on cell lysates collected from 12 to 60 h. In contrast to uninfected cells, SIRT1 protein expression demonstrated a clear time-dependent pattern. Notably, SIRT1 protein levels were upregulated between 24 and 60 hpi compared with the uninfected cells (Figure 1C).

As p53 serves as a major non-histone substrate of SIRT1, we assessed its expression at both the transcriptional and protein levels, as well as its acetylation status in parallel. MVC infection caused a gradual increase in p53 mRNA, which became significantly higher than that of the uninfected cells from 24 to 60 h (Figure 1B). Generally, p53 protein accumulated progressively over time, exhibiting an upward expression trend (Figure 1C). Although acetylated p53 (Ace-p53) was markedly elevated during early infection, this increase was not sustained in later periods (Figure 1C), diverging from the continued rise of total p53. This discrepancy indicates that, beyond acetylation, other post-translational modifications (such as phosphorylation) also contribute to p53 regulation during this process.

### 3.2. Nuclear Relocalization of SIRT1 and Its Colocalization with VP2 and p53

**Figure 2:**
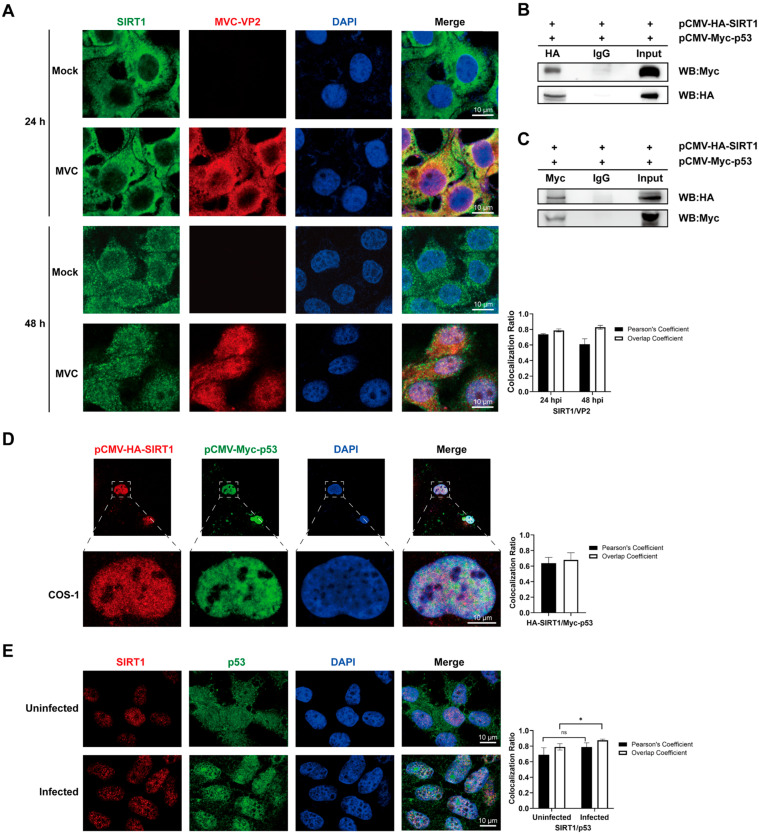
Immunofluorescence analysis of SIRT1 expression and its interaction with p53. (**A**) Subcellular localization of SIRT1 and MVC-VP2. WRD cells were subjected to mock infection or MVC infection at an MOI of 2, followed by immunostaining with anti-SIRT1 antibody (green), anti-VP2 antibody (red, as an indicator of MVC infection), and 4′,6-diamidino-2-phenylindole (DAPI, blue, for nuclear counterstaining) at 24 and 48 hpi. (**B**,**C**) Verification of the interaction between SIRT1 and p53 was performed by means of co-immunoprecipitation (Co-IP) assay. (**B**) COS-1 cells were transfected with pCMV-HA-SIRT1 and pCMV-Myc-p53 expression plasmids for 48 h. Total cellular lysates were prepared and subjected to immunoprecipitation (IP) with 3 μg anti-HA antibody or 3 μg rabbit IgG (isotype control). The immunoprecipitates and input lysates were analyzed by means of Western blotting (WB) using anti-Myc antibody. (**C**) Reciprocal Co-IP assay: total lysates from transfected COS-1 cells were immunoprecipitated with 3 μg anti-Myc antibody or 3 μg mouse IgG (isotype control), followed by WB detection with anti-HA antibody. (**D**) Colocalization of SIRT1 and p53 in transfected COS-1 cells. COS-1 cells were co-transfected with pCMV-HA-SIRT1 and pCMV-Myc-p53 plasmids for 48 h; the cells were fixed, permeabilized, and subjected to immunofluorescence (IF) staining. HA-SIRT1 was probed with anti-HA antibody and visualized with Alexa Fluor 594-conjugated secondary antibody (red), Myc-p53 with anti-Myc antibody, and Alexa Fluor 488-conjugated secondary antibody (green), and cell nuclei were counterstained with DAPI (blue). Images were acquired using a confocal laser scanning microscope (CLSM). (**E**) Immunofluorescence staining showing the overlap of endogenous SIRT1 and p53. WRD cells were infected with MVC for 48 h, then fixed, permeabilized, and subjected to IF staining with anti-SIRT1 antibody (red fluorescence) and anti-p53 antibody (green fluorescence); nuclei were counterstained with DAPI (blue). Colocalization signals were observed using a CLSM. Scale bars: 10 μm. Values represent the mean ± SD from three independent experiments. Comparisons were performed using Student’s *t*-tests. * *p* < 0.05. For colocalization analysis, Pearson’s correlation coefficient and overlap coefficient were calculated (shown on the right of each panel). ns: not significant.

Dynamic redistribution of SIRT1’s subcellular localization in response to stressful stimuli, including viral infections and autophagy, has been revealed in previous studies—and this shift is critical for its regulatory function in cellular homeostasis [36,37,38]. To investigate whether MVC infection alters SIRT1’s spatial distribution in WRD cells, immunofluorescence (IF) analysis was performed. Our results showed that, under normal conditions, SIRT1 exhibited predominant cytoplasmic and perinuclear localization at 24 h, progressing to perinuclear accumulation by 48 h in mock-infected cells. Upon MVC infection, the viral capsid protein VP2 displayed comparable cytoplasmic/perinuclear distribution at 24 hpi. Notably, SIRT1 underwent pronounced nuclear translocation at 48 hpi, accompanied by SIRT1-VP2 colocalization (Figure 2A). To further investigate whether SIRT1 participates in host regulatory pathways during infection, we assessed its interaction with p53. Co-immunoprecipitation assays in COS-1 cells co-expressing HA-SIRT1 and Myc-p53 demonstrated a specific and reciprocal association between the two proteins (Figure 2B,C). Subcellular localization analysis further demonstrated that overexpressed SIRT1 and p53 colocalized predominantly in the nucleus (Figure 2D). Moreover, endogenous proteins in WRD cells exhibited similar nuclear colocalization (Figure 2E), suggesting this interaction occurs under physiological conditions. Importantly, MVC infection enhanced this overlap, indicating that viral infection enhances SIRT1-p53 association. Collectively, these findings demonstrate that MVC infection promotes nuclear relocalization of SIRT1 and facilitates the formation of a SIRT1–p53 complex, providing a mechanistic basis for SIRT1-mediated modulation of p53 during viral infection.

### 3.3. Modulation of the SIRT1/p53 Axis Enhances Cell Viability and Promotes Viral Replication in MVC-Infected Cells

**Figure 3:**
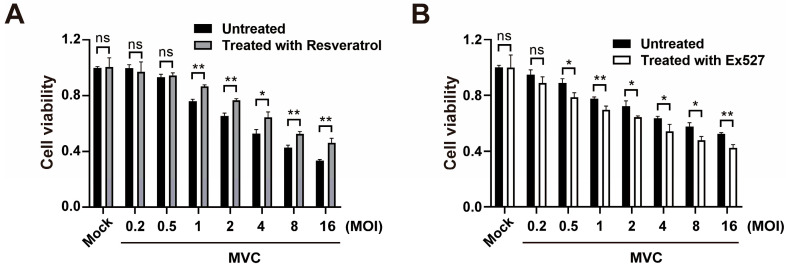
Effects of SIRT1 modulators on cell viability during MVC infection. WRD cells were treated with 20 μM resveratrol (**A**) or 50 μM Ex527 (**B**) and infected with MVC of different MOIs. Cell viability was assessed by using CCK-8 reagent at 72 hpi. Data are shown as means ± standard deviations (*n* = 3 biological replicates, each with 3 technical replicates). *p* values were determined using Student’s *t*-test. * *p* < 0.05; ** *p* < 0.01; ns: not significant.

To evaluate the effects of SIRT1 modulators on cell viability during MVC infection, WRD cells were treated with the SIRT1 activator resveratrol or inhibitor Ex527 and subsequently infected with MVC at increasing multiplicities of infection (MOI). As shown in Figure 3A, MVC infection resulted in a dose-dependent reduction in cell viability, with decreases evident at MOIs of 2 or greater. Resveratrol treatment did not significantly affect cell viability at low MOIs (0.2 and 0.5); however, at higher MOIs (1–16), resveratrol significantly protected cells from MVC-induced cytotoxicity. Similarly, treatment with the SIRT1 inhibitor Ex527 had no significant effect on cell viability at a low MOI (0.2) (Figure 3B). Conversely, at higher MOIs (1–16), Ex527 significantly potentiated MVC-induced cell death, resulting in further reduced viability compared with untreated cells.

**Figure 4:**
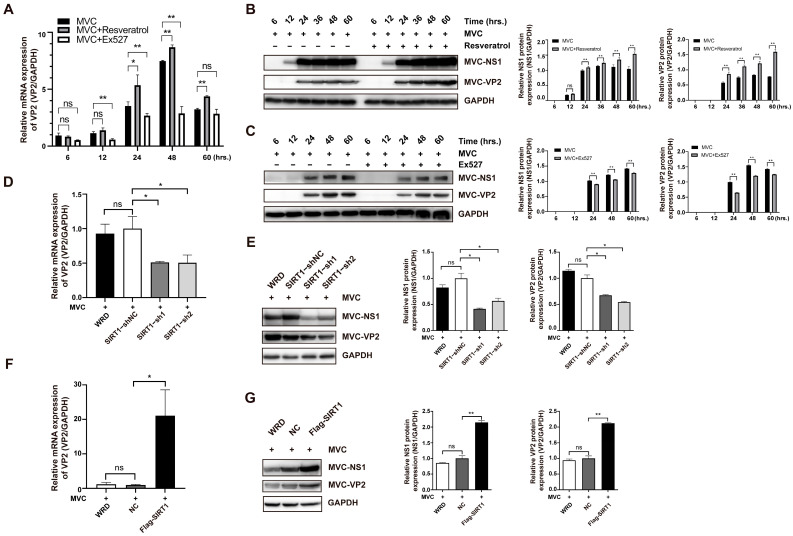
SIRT1 positively regulates MVC gene expression and viral protein accumulation. (**A**) Relative mRNA expression of MVC VP2 in cells treated with resveratrol or Ex527 at 6–60 hpi. VP2 mRNA levels were quantified by means of RT-qPCR and normalized to GAPDH. (**B**,**C**) Protein expression levels of MVC NS1 and VP2 in cells treated with resveratrol (**B**) or Ex527 (**C**) following MVC infection at 6–60 hpi. Representative Western blotting images are shown on the left, and corresponding densitometric quantification normalized to GAPDH is shown on the right. (**D**) Real-time qPCR analysis of MVC VP2 mRNA levels in WRD stable cell lines with SIRT1 depletion (SIRT1–sh1/sh2) or their control cell lines (SIRT1–shNC) at 24 hpi. (**E**) Western blot analysis and quantitative analyses of MVC NS1 and VP2 in SIRT1-depleted cells following MVC infection. (**F**) Relative mRNA expression of MVC VP2 in WRD stable cell lines with SIRT1 overexpression (Flag-SIRT1) or control cell lines following MVC infection at 48 h. (**G**) Western blot analysis of MVC NS1 and VP2 in SIRT1 overexpressing cells following MVC infection. Quantifications are shown on the right. Data are shown as means ± standard deviations from three independent experiments. *p* values were determined using Student’s *t*-test. * *p* < 0.05; ** *p* < 0.01; ns: not significant.

To examine the role of SIRT1 in MVC replication, we first assessed the impact of pharmacological modulation of SIRT1 on viral gene expression. WRD cells were treated with resveratrol (a SIRT1 agonist) or the inhibitor Ex527 following MVC infection at an MOI of 2. RT-qPCR analysis revealed that resveratrol treatment significantly increased MVC VP2 mRNA levels at 24–60 hpi compared with mock-treated controls, whereas Ex527 treatment markedly reduced VP2 mRNA expression at 12–48 hpi (Figure 4A). Consistent with these findings, Western blot analysis showed that resveratrol treatment enhanced the protein expression of MVC NS1 and VP2 in a time-dependent manner, with significantly elevated levels observed from 24 to 60 hpi compared with mock-treated controls (Figure 4B). In contrast, Ex527 treatment resulted in a pronounced reduction in NS1 and VP2 protein levels at 24–60 hpi (Figure 4C).

To verify these pharmacological results, we next examined the effects of genetic manipulation of SIRT1 on MVC replication. SIRT1 expression was knocked down using two distinct SIRT1-targeting shRNA expression vectors. RT-qPCR analysis showed that knockdown of SIRT1 significantly decreased VP2 mRNA levels by ~49% (0.51 ± 0.02 vs. 1.00 ± 0.17) and ~50% (0.50 ± 0.11 vs. 1.00 ± 0.17), respectively, compared with the negative control (Figure 4D). Correspondingly, Western blot analysis revealed that SIRT1 knockdown markedly reduced NS1 and VP2 protein levels (NS1: ~59% or ~43%; VP2: ~33% or ~46%) compared with the SIRT1–shNC group (Figure 4E). Conversely, SIRT1 overexpression increased VP2 mRNA levels by 20-fold compared with the negative control following MVC infection (Figure 4F). This upregulation was further validated at the protein level, as Western blot analysis demonstrated that SIRT1 overexpression substantially elevated NS1 and VP2 levels by more than two-fold (NS1: 2.15-fold; VP2: 2.12-fold) (Figure 4G). Collectively, these results demonstrate that pharmacological activation or genetic overexpression of SIRT1 promotes MVC gene expression and protein accumulation, whereas inhibition or knockdown of SIRT1 suppresses MVC replication.

### 3.4. Pharmacological Modulation of SIRT1 on Cell Cycle Progression and Apoptosis Following MVC Infection

**Figure 5:**
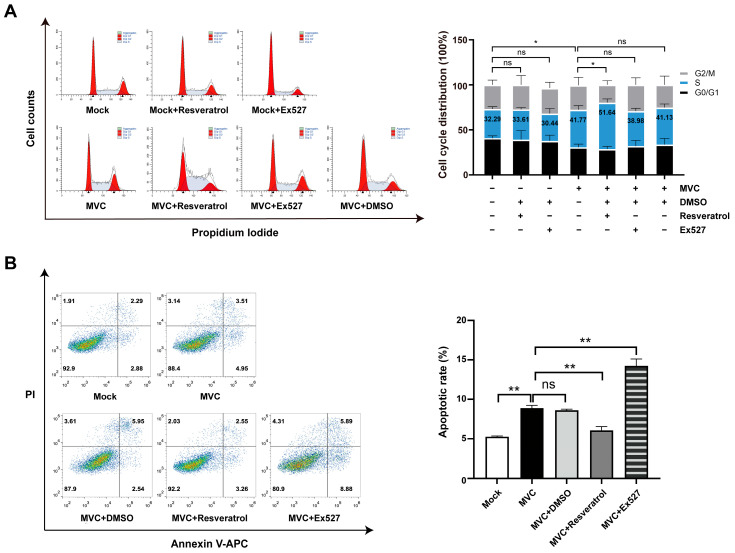
Analysis of cell cycle distribution and apoptosis following SIRT1 modulation in MVC-infected cells. (**A**) SIRT1 modulation alters cell cycle distribution in MVC-infected cells. WRD cells were treated with 20 μM resveratrol or 50 μM Ex527 and infected with MVC at an MOI of 2. After 48 h of infection, cells were harvested, fixed in 75% ethanol overnight at 4 °C, and stained with Propidium Iodide (PI) for flow cytometry analysis. DNA content of the indicated groups was analyzed using the software ModFit. Quantification of cell cycle phases is presented on the right. The blue bars represent the proportion of cells in S phase, and statistical analysis of S phase distribution is shown. (**B**) Opposing effects of SIRT1 activation and inhibition on MVC-induced cell death. Cells were processed as described above and then subjected to Annexin V-APC/PI double staining. Apoptosis was immediately assessed by means of flow cytometry. The APC−/PI− population represents live cells, APC+/PI− cells are early apoptotic cells, and the APC+/PI+ population indicates late apoptosis. The apoptotic cell population of each group was analyzed using the software FlowJo. Quantitative analysis of cell apoptosis rate in WRD cells treated with resveratrol or Ex527 is shown on the right. Data are shown as means ± standard deviations from 3 independent experiments. *p* values were determined using Student’s *t*-test. * *p* < 0.05; ** *p* < 0.01; ns: not significant.

We next sought to determine how SIRT1 enhances MVC replication. We first asked whether SIRT1-mediated viral promotion was associated with alterations in cell cycle distribution or apoptosis. Qiu et al. demonstrated that S-phase accumulation was detected during MVC infection, which can slow down host cellular DNA replication and enable MVC to utilize host polymerase and replication factors to facilitate its own DNA replication [39]. Given that cell cycle arrest and apoptosis are closely linked to viral replication efficiency and cell survival, we assessed cell cycle distribution and apoptosis under SIRT1 modulation following MVC infection. With DNA staining, we found that MVC infection triggered a distinct cell accumulation in the S-phase of WRD cells (41.77% versus 32.29% in uninfected cells). To assess the contribution of SIRT1 activity to this process, we examined the effects of resveratrol and Ex527 on MVC-induced changes on cell cycle distribution (Figure 5A). Resveratrol treatment increased the S-phase arrest by 24% (51.64% ± 4.13 vs. 41.77% ± 4.48) compared with MVC-infected controls, while Ex527 treatment resulted in an S-phase population of 38.98%, showing no significant effect. These findings demonstrate that pharmacological activation of SIRT1 induces prolonged S-phase cell cycle arrest in MVC-infected cells. We next evaluated apoptosis following pharmacological modulation of SIRT1. As shown in Figure 5B, MVC infection significantly increased the apoptotic rate from 5.27% (±0.11) to 8.88% (±0.37), representing a 1.69-fold increase over the uninfected control. Resveratrol treatment attenuated this effect, lowering apoptosis to 6.06% (±0.49)—a reduction of nearly one-third relative to MVC-infected cells. Conversely, Ex527 intensified cell death to 14.24% (±0.88), approximately 1.6 times the level observed in infected controls. These divergent pharmacological manipulations establish SIRT1 as a pivotal modulator of MVC-induced apoptosis.

### 3.5. Modulation of SIRT1 Affects Apoptosis-Related Protein Expression and Thereby Influences Viral Production

**Figure 6:**
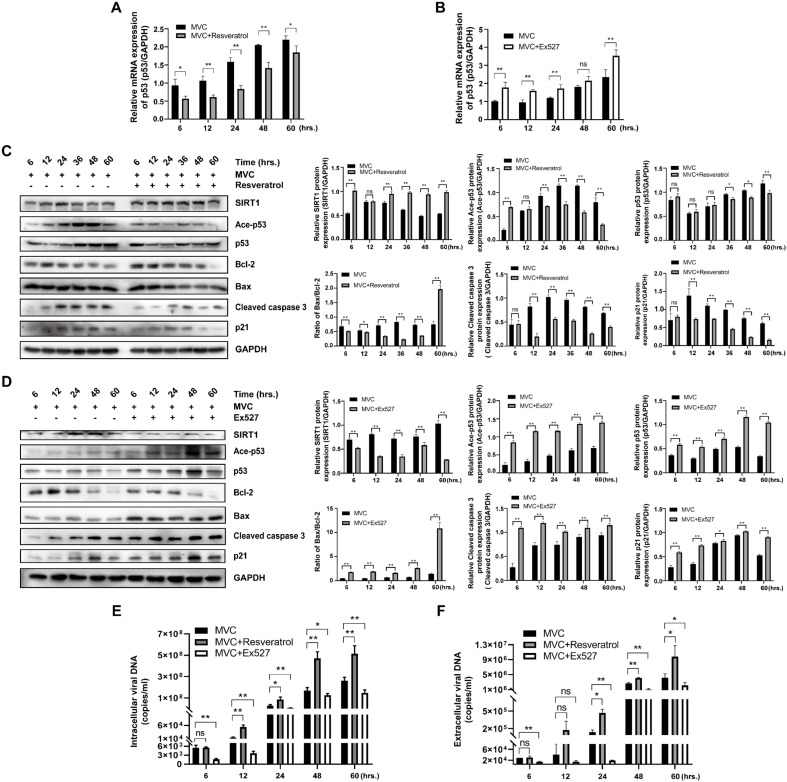
Effects of SIRT1 activation and inhibition on apoptosis-related molecules and viral production. MVC-infected cells were treated with resveratrol or Ex527, with untreated infected cells as the control. (**A**,**B**) RT-qPCR was performed to assess p53 mRNA expression in MVC-infected cells with treatment of 20 μM resveratrol (**A**) or 50 μM Ex527 (**B**). (**C**,**D**) Western blot analysis was conducted to examine the expression of apoptosis-related molecules in WRD cells infected by MVC following treatment with resveratrol (**C**) or Ex527 (**D**). Densitometric quantification of the indicated band intensities is presented on the right, with protein levels normalized to GAPDH. (**E**,**F**) Viral production was evaluated by quantifying intracellular (**E**) and extracellular (**F**) viral loads using absolute qPCR. Data are shown as means ± standard deviations from 3 independent experiments. Statistical significance was determined using Student’s *t*-test. * *p* < 0.05; ** *p* < 0.01; ns: not significant.

Having established that SIRT1 modulates both cell cycle progression and apoptosis in MVC-infected cells, we next investigated the molecular mechanisms underlying this regulation, specifically examining apoptosis-related protein expression and its impact on viral production. RT-qPCR analysis showed that treatment with the SIRT1 activator resveratrol suppressed p53 mRNA levels from 6 to 60 hpi compared with the MVC-infected control (Figure 6A). At the protein level, Western blot analysis revealed that resveratrol treatment significantly increased SIRT1 protein expression and was accompanied by a substantial decrease in acetylated p53 (Figure 6C). Consistently, the expression levels of pro-apoptotic proteins, including p53, Bax, Cleaved caspase 3, and p21 exhibited a downward trend, and the anti-apoptotic Bcl-2 was elevated following resveratrol intervention (Figure 6C). However, cells were already in a late apoptotic state at 60 hpi; consequently, resveratrol treatment failed to exert a protective effect or restore Bcl-2 expression. Given that resveratrol attenuated apoptosis in MVC-infected cells, we next investigated whether this protective effect correlated with altered viral production. Intracellular and extracellular viral DNA levels were subsequently quantified. Resveratrol treatment significantly increased intracellular viral DNA copy numbers from 12 to 60 hpi compared with the MVC-infected control (Figure 6E). Consistently, extracellular viral DNA levels were also markedly elevated in resveratrol-treated cells from 24 to 60 hpi (Figure 6F).

To confirm that these effects were specifically mediated by SIRT1, we used Ex527 to inhibit SIRT1 activity. In contrast to resveratrol treatment, Ex527 enhanced the upregulation of p53 transcripts at 6–24 hpi and 60 hpi (Figure 6B). Ex527 treatment attenuated SIRT1 expression, restored p53 acetylation, and promoted apoptosis, as evidenced by upregulation of p53, Bax, Cleaved caspase 3 and p21, and downregulation of Bcl-2 from 6–60 hpi compared with the infected control group (Figure 6D). Consistent with the observed enhancement of apoptosis, Ex527 treatment reduced intracellular viral DNA levels from 6 to 60 hpi and concomitantly decreased extracellular viral release at 6 hpi and 24–60 hpi compared with the infected control (Figure 6E,F).

Collectively, these results demonstrate that SIRT1 activation by resveratrol suppresses MVC-induced apoptosis and p53-associated signaling, yet enhances MVC production. Conversely, SIRT1 inhibition by Ex527 exacerbates apoptotic and p53-mediated responses while restricting viral spread. Thus, SIRT1 functions as a molecular switch governing the trade-off between host cell viability and viral production during MVC infection.

### 3.6. SIRT1 Knockdown Attenuates S-Phase Cell Cycle Arrest, Promotes Caspase-Dependent Apoptosis and Restricts Viral Propagation

**Figure 7:**
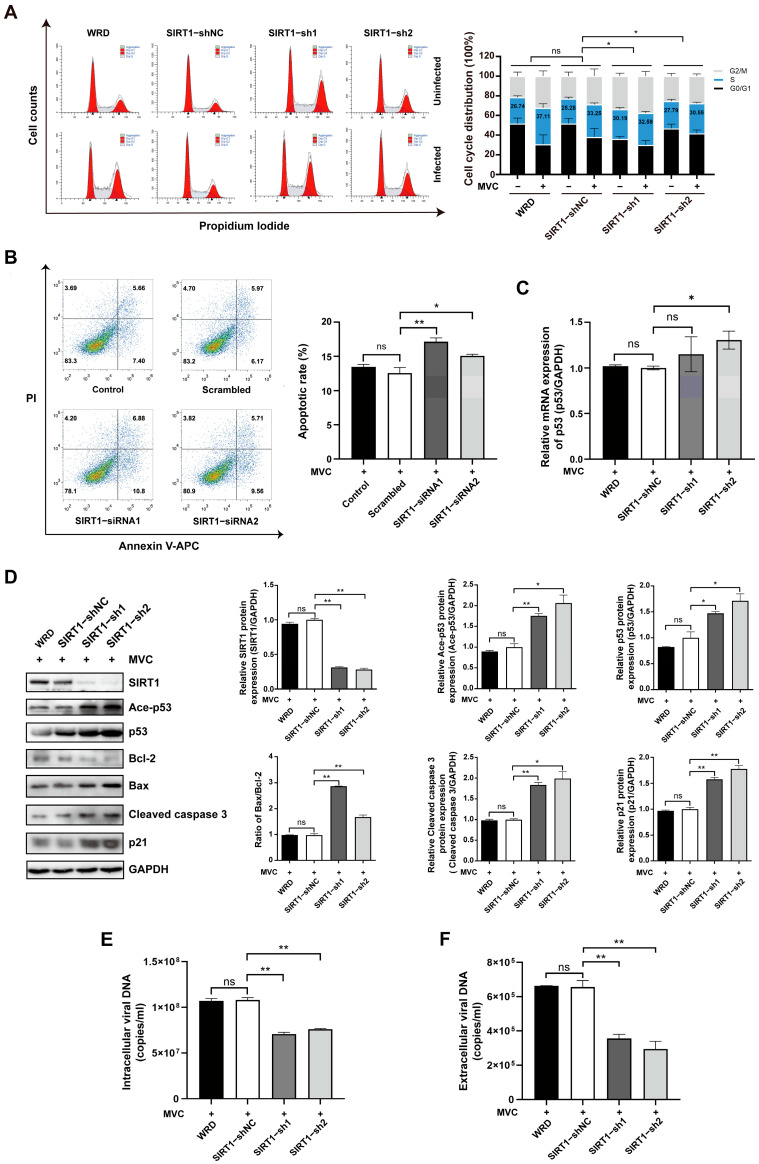
Knockdown of SIRT1 alleviates MVC-induced S-phase arrest and enhances apoptotic cell death, consequently restricting viral propagation. (**A**) SIRT1 depletion decreased MVC-induced S-phase arrest. WRD cells stably expressing control shRNA or SIRT1-targeting shRNA were infected with MVC and subjected to cell cycle analysis by means of PI staining and flow cytometry at 24 hpi. The right panel shows quantification of cell cycle phases. Blue columns show the relative abundance of cells in S phase, with corresponding statistical analysis of S phase distribution. (**B**) SIRT1 silencing enhanced MVC-triggered apoptosis. WRD cells transfected with control siRNA or SIRT1 siRNA for 48 h were infected with MVC (MOI = 2) for an additional 48 h, apoptotic fractions were quantified by means of Annexin-V/PI flow cytometry. (**C**) SIRT1 knockdown upregulated p53 transcript abundance in MVC-infected cells. Total RNA was harvested and analyzed by means of qRT–PCR. (**D**) Impact of SIRT1 depletion on apoptosis-associated proteins. Whole-cell lysates prepared at 24 hpi were immunoblotted for Ace-p53, p53, Cleaved caspase 3, Bax, Bcl-2, p21, and GAPDH (loading control). (**E**) Intracellular virion DNA was diminished upon SIRT1 knockdown. Hirt DNA extracted from control shRNA- or SIRT1-specific shRNA-expressing cells at 24 hpi was detected by means of absolute quantitative real-time PCR using MVC-specific primers, and genome copy numbers were calculated against a standard curve. (**F**) Knockdown of SIRT1 reduced the release of progeny virions. Extracellular virions in clarified culture supernatants collected at 24 hpi were diluted and quantified by means of absolute qPCR. Data are shown as means ± standard deviations from 3 independent experiments. *p* values were determined using Student’s *t*-test. * *p* < 0.05; ** *p* < 0.01; ns: not significant.

To systematically elucidate the functional role of SIRT1 during MVC infection, we performed lentiviral-mediated knockdown using two independent shRNAs (SIRT1−sh1/sh2). Following MVC infection, SIRT1-depleted cells exhibited S-phase populations of 32.59% (±1.74) and 30.55% (±1.26), corresponding to increases of only 2.39% and 2.76% relative to uninfected controls. In contrast, shNC control cells displayed a 7.97% (33.25% vs. 25.28%) increase in S-phase cells upon infection. Consequently, SIRT1 depletion reduced MVC-induced S-phase arrest by 70% (2.39% ± 1.89 vs. 7.97% ± 0.46) and 65% (2.76% ± 2.12 vs. 7.97% ± 0.46), respectively (Figure 7A). To avoid GFP interference in fluorescein-based flow cytometric apoptosis assays, SIRT1 expression was silenced using non-fluorescent siRNA targeting the same sequence as the lentiviral shRNA. Consistent with Ex527 treatment, siRNA-mediated SIRT1 depletion increased the apoptotic cell population from 12.58% ± 0.80 (scrambled siRNA) to 17.16% ± 0.54 (36% increase) and 15.05% ± 0.24 (20% increase), confirming that SIRT1 knockdown exacerbates MVC-induced apoptosis (Figure 7B). We further examined the effect of SIRT1 silencing on the expression of apoptosis-related molecules. As expected, p53 mRNA levels increased 1.3-fold in the SIRT1−sh2 group (Figure 7C). At the protein level, Western blot analysis showed that knocking down SIRT1 induced significant upregulation of the pro-apoptotic proteins, including Ace-p53, p53, Bax, Cleaved caspase 3, and p21, while downregulating the protein Bcl-2 (Figure 7D). Consistent with these findings, intracellular virion levels were reduced by 34% and 29% in SIRT1 shRNA-transduced cells (Figure 7E), and the release of viral progeny into the culture medium was decreased by 46% and 55% (Figure 7F).

### 3.7. SIRT1 Overexpression Promotes S-Phase Cell Cycle Arrest, Reduces Apoptotic Protein Expression and Augments Viral Yields

**Figure 8:**
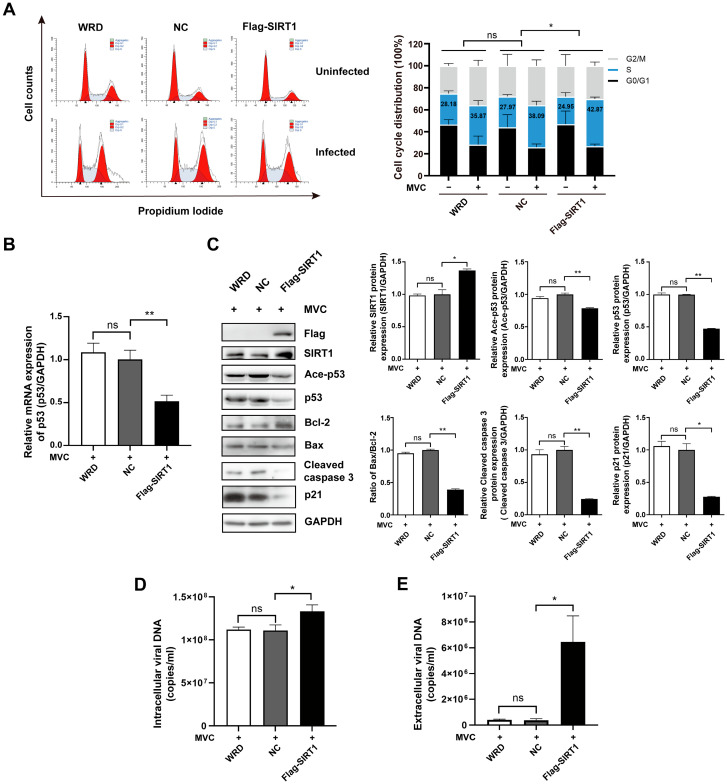
Overexpression of SIRT1 enforces S-phase arrest, reduces apoptotic proteins, and promotes MVC replication. (**A**) SIRT1 overexpression promoted S-phase arrest. WRD cells transduced with lentiviruses containing the SIRT1 overexpressing vector or empty vector were infected with MVC (MOI = 2) and analyzed by means of PI staining and flow cytometry at 48 hpi. Representative histograms from three independent experiments are shown, with corresponding quantification of cell cycle phases on the right. Blue columns represent S phase cell proportions, and statistical analysis of S phase distribution is displayed. (**B**) RT-PCR analysis of p53 mRNA expression in SIRT1-overexpressing cells and control cells. (**C**) Western blot analysis of apoptotic proteins in cells transduced with the SIRT1 overexpressing vector or empty vector. Cell lysates were prepared at 48 hpi and immunoblotted for Ace-p53, p53, Bax, Bcl-2, Cleaved caspase 3, p21, and GAPDH (loading control). (**D**) SIRT1 overexpression elevated intracellular viral particles. Hirt DNA was extracted at 48 hpi and determined by means of absolute qPCR; the viral DNA copies were calculated using a standard curve. (**E**) SIRT1 overexpression boosted the release of viral progeny in the culture medium. The clarified supernatants collected at 48 hpi were diluted and quantified by means of absolute qPCR. Data are shown as means ± standard deviations from 3 independent experiments. *p* values were determined using Student’s *t*-test. * *p* < 0.05; ** *p* < 0.01; ns: not significant.

To evaluate the impact of SIRT1 gain-of-function on MVC infection, WRD cells were transduced with lentiviral particles encoding Flag-tagged SIRT1 or the empty vector control, followed by MVC infection at an MOI of 2. At 48 h post-infection, cells were harvested for cell cycle analysis via PI staining and flow cytometry. Following MVC infection, SIRT1-overexpressing cells displayed an S-phase population of 42.87% (±1.75), corresponding to a 17.92% increase relative to uninfected controls. By comparison, control NC cells showed 38.09% (±3.71) in the S-phase, representing a 10.12% increase upon infection. Consequently, SIRT1 overexpression exacerbated MVC-induced S-phase arrest by 77% (17.92% ± 1.75 vs. 10.12% ± 3.71). (Figure 8A). Quantitative RT-PCR demonstrated that SIRT1 overexpression downregulated p53 mRNA levels by 48% (Figure 8B). Immunoblotting of infected-cell lysates showed that SIRT1 overexpression caused a reduction in Ace-p53, p53, Bax, Cleaved caspase 3, and p21, but increased Bcl-2 levels (Figure 8C), reinforcing its role as a negative regulator of p53 acetylation and downstream apoptosis during MVC infection. Hirt DNA quantification revealed a modest 1.2-fold increase in intracellular viral genomes (Figure 8D), while extracellular virions in the culture supernatant rose markedly by 17.5-fold (Figure 8E). These findings collectively demonstrate that SIRT1 serves as a proviral factor, enhancing both MVC genome replication and progeny release.

## 4. Discussion

Viruses have evolved multiple strategies to utilize host cellular regulatory networks to support viral replication. For parvoviruses with limited coding capacity, genome replication largely depends on the manipulation of host cell cycle regulation, stress responses, and cell death pathways. In this context, host epigenetic regulators that integrate DNA damage repair, cell cycle control, and viral replication are increasingly recognized as important regulatory nodes in virus–host interactions. It has been previously reported that MVC infection induces pronounced cytopathic effects in permissive WRD cells, characterized by mitochondria-mediated apoptosis [21]. Notably, the induction of apoptosis was strictly dependent on viral genome replication, indicating a close coupling between MVC replication and host cell death. However, the host regulatory mechanisms coordinating MVC replication with apoptotic signaling remained unclear. In the present study, we identify SIRT1 as an important host factor involved in this process and provide evidence that MVC infection dynamically modulates the SIRT1/p53 signaling axis. Previous studies have elucidated the regulatory effects of SIRT1 in widespread viral infections, including hepatitis B virus (HBV) [40], human papillomavirus (HPV) [32], human immunodeficiency virus (HIV) [31], enterovirus 71 (EV71) [36], and severe acute respiratory syndrome coronavirus 2 (SARS-CoV-2) [41]. Our study addresses this research gap by revealing the dynamic alterations of the SIRT1-p53 deacetylation pathway during MVC infection, thereby providing a novel perspective on the epigenetic regulatory mechanisms governing MVC–host cell interactions.

SIRT1 expression levels are highly context-dependent, exhibiting substantial variation across diverse viral infections and host systems. For example, SIRT1 is markedly upregulated in EV71-infected human rhabdomyosarcoma (RD) and neuroblastoma (SK-N-SH) cells [36]. RSV stimulation increases SIRT1 expression in mouse lung tissue and dendritic cells [28]. In HBV models, SIRT1 levels are elevated in HepG2.2.15 and HepG2-HBV1.1 cells, and HBV-expressing adenovirus infection of human primary hepatocytes (PHH) also increases SIRT1 protein expression relative to the control adenovirus [30]. By contrast, some viral infections promote apoptosis through increased ROS production and reduced SIRT1 expression; notably, porcine parvovirus infection downregulates SIRT1 transcripts in PK-15 cells at 24 hpi [42]. These observations indicate that SIRT1 regulation during infection is dynamic and virus dependent. In addition to expression changes, SIRT1 subcellular localization is dynamic and can shift between the cytoplasm and nucleus in response to cellular stress [36,43]. In our system, SIRT1 predominantly localized to the cytoplasm and perinuclear region in untreated cells, whereas MVC infection promoted SIRT1 nuclear translocation and revealed colocalization with MVC VP2. Together, these findings support the involvement of SIRT1 in MVC infection and suggest that MVC may actively reprogram SIRT1 localization to facilitate infection-associated processes in the nucleus.

Protein acetylation is a prevalent post-translational modification implicated in transcriptional regulation and cellular responses to infection, and increasing evidence highlights its importance in controlling viral replication. In influenza virus infection, acetylation of the viral nucleoprotein (NP) at Lys31 and Lys90 by the host acetyltransferases GCN5 and PCAF exerts opposing effects on viral replication [44]. Beyond direct modification of viral proteins, host cells may also modulate the acetylation status of host substrates to restrict infection; for example, HDAC6-mediated deacetylation of microtubulin affects multiple stages of influenza A virus entry, replication, transport, and release, thereby inhibiting infection [45]. SIRT1 can influence viral replication by regulating key host molecules and, in some contexts, by directly deacetylating viral proteins [26,27,30,31]. SIRT1-dependent regulation of p53 acetylation has also been described during vesicular stomatitis virus (VSV) replication [46]. In our study, we detected substantial changes in acetylated p53 in MVC-infected cells, confirmed the interaction between SIRT1 and p53, and showed that SIRT1 affects MVC replication by modulating p53 acetylation.

MVC-induced cell cycle arrest has been reported to occur mainly at the G2/M phase [21], whereas other studies suggest that MVC can also trigger S-phase arrest, slowing host DNA replication and recruiting cellular DNA replication factors for viral DNA synthesis [39]. Our observations support the latter finding, with MVC infection producing a pronounced S-phase arrest in WRD cells. While SIRT1-mediated deacetylation of p53 is classically associated with G1 checkpoint release [47], our findings revealed enhanced S-phase arrest upon SIRT1 activation. This apparent paradox can be explained by the unique cellular context of viral infection. We propose that SIRT1 inactivates p53 to prevent apoptosis that would otherwise eliminate S-phase-arrested cells, thereby preserving a cellular environment conducive to viral replication. Concurrently, SIRT1 maintains S-phase arrest through p53-independent mechanisms, potentially involving ATM-SMC1 signaling [39]. By preventing apoptosis while sustaining S-phase arrest, SIRT1 creates a favorable environment for viral replication, explaining why SIRT1 inhibition leads to increased apoptosis and reduced viral titers. Mechanistically, p53 can promote apoptosis by increasing mitochondrial membrane permeability through interactions with Bcl-2 family proteins, leading to Bax or Bak activation [48,49]. p53 response elements are also present in the promoters of anti-apoptotic genes such as *Bcl-2* and *survivin*, both of which are negatively regulated by p53 [50,51]. In line with these established pathways, SIRT1 overexpression or resveratrol induction increased SIRT1 levels and reduced acetylated p53, accompanied by decreased expression of p53, p21, Bax, and Cleaved caspase 3, together with increased Bcl-2 expression. Conversely, these changes were reversed by SIRT1 silencing or treatment with the SIRT1 inhibitor Ex527. However, Ex527 did not significantly alleviate S-phase arrest; this outcome may reflect compensatory activation of other sirtuin family members and warrants further investigation.

Although our study demonstrates that MVC infection activates the SIRT1/p53 signaling pathway and exerts significant effects on viral replication and virus-induced cytopathic effects, several limitations should be acknowledged. During MVC infection, we observed a sustained upregulation of SIRT1 accompanied by dynamic changes in p53 acetylation; however, whether SIRT1 directly regulates additional host or viral substrates beyond p53 during MVC infection remains unclear. Further studies are required to clarify the molecular basis of these interactions. Resveratrol is widely recognized as a SIRT1 activator that regulates cellular metabolism and stress responses, yet it is important to note that resveratrol also exhibits pleiotropic SIRT1-independent effects, including antioxidant activity, modulation of other signaling pathways, and interactions with various cellular targets. Therefore, the observed phenotypes induced by resveratrol in our study may not be mediated exclusively through SIRT1 activation. While our findings suggest a significant role for SIRT1 in mediating the observed effects, we acknowledge that additional mechanisms may also contribute to the cellular response of resveratrol. The relative contributions of SIRT1-dependent and -independent pathways will be required to achieve a more comprehensive understanding of resveratrol’s actions in the context of MVC infection.

In summary, this study demonstrates that SIRT1 plays an important regulatory role in MVC infection by modulating p53 acetylation and downstream stress responses. We propose a model in which MVC-induced SIRT1 activation suppresses p53 acetylation, attenuates apoptosis, and promotes cell cycle conditions favorable for viral genome replication. These findings establish a direct link between host epigenetic regulation and the MVC life cycle and suggest that targeting the SIRT1–p53 axis may represent a potential strategy for controlling bocaparvovirus infection.

## Data Availability

The original contributions presented in this study are included in the article. Further inquiries can be directed to the corresponding author.
